# Temporal dynamics of in-situ fiber-adherent bacterial community under ruminal acidotic conditions determined by 16S rRNA gene profiling

**DOI:** 10.1371/journal.pone.0182271

**Published:** 2017-08-01

**Authors:** Renee M. Petri, Poulad Pourazad, Ratchaneewan Khiaosa-ard, Fenja Klevenhusen, Barbara U. Metzler-Zebeli, Qendrim Zebeli

**Affiliations:** 1 Institute of Animal Nutrition and Functional Plant Compounds, Department for Farm Animals and Veterinary Public Health, University of Veterinary Medicine Vienna, Vienna, Austria; 2 Clinic for Swine, Department for Farm Animals and Veterinary Public Health, University of Veterinary Medicine Vienna, Vienna, Austria; University of Illinois, UNITED STATES

## Abstract

Subacute rumen acidotic (SARA) conditions are a consequence of high grain feeding. Recent work has shown that the pattern of grain feeding can significantly impact the rumen epimural microbiota. In a continuation of these works, the objective of this study was to determine the role of grain feeding patterns on the colonization and associated changes in predicted functional properties of the fiber-adherent microbial community over a 48 h period. Eight rumen-cannulated Holstein cows were randomly assigned to interrupted or continuous 60%-grain challenge model (n = 4 per model) to induce SARA conditions. Cows in the continuous model were challenged for 4 weeks, whereas cows of interrupted model had a 1-wk break in between challenges. To determine dynamics of rumen fiber-adherent microbial community we incubated the same hay from the diet samples for 24 and 48 h in situ during the baseline (no grain fed), week 1 and 4 of the continuous grain feeding model as well as during the week 1 following the break in the interrupted model. Microbial DNA was extracted and 16SrRNA amplicon (V3-V5 region) sequencing was done with the Illumina MiSeq platform. A significant decrease (*P* < 0.001) in fiber-adherent rumen bacterial species richness and diversity was observed at the end of a 4 week continuous SARA challenge in comparison to the baseline. A total of 159 operational taxonominc units (OTUs) were identified from the microbial population representing > 0.1% relative abundance in the rumen, 18 of which were significantly impacted by the feeding challenge model. Correlation analysis of the significant OTUs to rumen pH as an indicator of SARA showed genus *Succiniclasticum* had a positive correlation to SARA conditions regardless of treatment. Predictive analysis of functional microbial properties suggested that the glyoxylate/dicarboxylate pathway was increased in response to SARA conditions, decreased between 24h to 48h of incubation, negatively correlated with propanoate metabolism and positively correlated to members of the *Veillonellaceae* family including *Succiniclasticum* spp. This may indicate an adaptive response in bacterial metabolism under SARA conditions. This research clearly indicates that changes to the colonizing fiber-adherent rumen microbial population and their predicted functional genes occur in both the short (48 h) and long term (4 wk) under both continuous and interrupted SARA challenge models.

## Introduction

The rumen microbial community is highly impacted by diet and host factors such as age and health status [1]. It is well understood that the replacement of fiber with more readily fermentable carbohydrates impacts these microorganisms and alters the dynamics of the rumen ecosystem [1, 2] leading to metabolic diseases such as subacute ruminal acidosis (SARA) [3, 4]. Subacute ruminal acidosis not only impacts feed efficiency but is associated with a large number of costly metabolic disorders and may also lead to systemic inflammation [5]. Most of the research looking at the impacts of SARA on the rumen microbial ecosystem assumes that the animal is continuously exposed to SARA conditions, and the course of exposure is relatively short lasting from a few days to 2 wk [4, 5, 6]. However, dairy herd cows can typically experience SARA conditions periodically throughout the lactation cycle related to diet changes with calving, transitioning and lactation [7]. Furthermore, these episodes are most likely to be transient in nature as animals self-regulate intake due to changes in rumen osmolarity, contractions, and passage rate [8]. However, comparisons between long-term and intermittent SARA models remain relatively under researched. Since fiber-adherent ruminal microorganisms require longer time for colonization and fermentation in the rumen [3], intermittent SARA conditions may be a greater challenge for the establishment of particle associated bacterial populations. Based on this we hypothesized that fiber-adherent microbial population would have less cellulolytic bacteria, decreased diversity and down-regulated carbohydrate metabolism within a 48 h period on a intermittent SARA model compared to a long term model which would allow for host and microbe adaptation.

## Materials and methods

### Animals and experimental design and challenge models

Animal handling and experimental procedures were approved by the institutional ethics committee of the University of Veterinary Medicine, Vienna and the Austrian authority according to §26 of Law for Animal Experiments, Tierversuchsgesetz 2012 –TVG (GZ 68.205/0093-II/3b/2013).

This study was conducted in part with investigations on ruminal pH and temperature profile [9], ruminal absorption [10] and rumen epimural microbiota [7]. In brief, eight ruminally cannulated (100 mm i.d. Bar Diamond, ID) non-lactating Holstein cows (BW: 710± 118 kg, age: 68 ± 20 mo, mean ± SD) were blocked by BW and randomly assigned to one of two feeding models for the induction of SARA (n = 4 per challenge model). These cattle had no previous exposure to SARA induction experiments. The experiment consisted of a 40-d measurement period (a 6-d baseline, a 6-d gradual adaptation, and a continuous or interrupted 28-d SARA challenge). The ingredient and chemical composition of the diets (forage-only and SARA diets; S1 Table) used in the current study and the challenge design have been previously published [9]. For a minimum of 2 wk prior to the start of the experiment, all cows were fed a forage mix consisting of hay and grass silage (1:1 DM basis) to allow for adaptation to the individualized feeding system (RIC system; Insentec B.V., Marknesse, The Netherlands). The same baseline forage diet was also provided during the interval challenge break. Transitioning to the SARA diet was done gradually (10% concentrate DM/day) over 6 d to a dietary composition of 60:40, concentrate to forage ratio (DM basis). The forage mix and concentrate were offered separately. Cows were provided access to the forage mix starting at 0800 h and the concentrate starting at 1000 h daily. Water was provided ad libitum. Cows were housed at the research farm of the University of Veterinary Medicine, Vienna, Austria during the experiment and were kept together in a loose-housing stable with straw-bedding.

The two SARA challenge models used were interrupted (INT) and continuous (CONT) SARA. During both challenges, the concentrate level was maintained at 60% (DM basis) and cows were restrictively fed. During the forage feeding periods the diet was offered at 1.5% of body weight (BW), whereas during adaptation and SARA periods the diet was provided at 2.0% of BW (DM basis). After the baseline and adaptation, cows in the CONT model remained on the SARA diet continuously for 28 d. For cows in the interrupted model, after baseline and adaptation, SARA diets were provided for 7 d followed by a 7 d break (forage only) and then a second SARA challenge for 14 d (a 2-d gradual adaptation and a 12-d full challenge). During the SARA challenge any orts of concentrate were delivered via the cannula (2.48 ± 3.66 kg DM/day) to ensure an equal dietary forage to concentrate ratio for all cows.

### Rumen pH, in situ incubation and sample collection

At the beginning of the experiment each cow received wireless ruminal pH sensors (smaXtec^®^, Graz, Austria) which were introduced directly through the rumen cannula to examine daily ruminal pH and temperature dynamics. More details regarding pH sensor measurements are previously described in [9]. Only mean ruminal pH, on day of sampling, in correlation to in situ bag microbial populations are examined in the present study. In situ incubation was performed twice for cows undergoing the interrupted challenge: during the baseline (4 d prior to adaptation; Base-I) and during the first week of the re-challenge after the break (5 d after the break; INT). In situ incubations were performed 3 times for cows in the CONT model: during the baseline (4 d prior to adaptation; Base-C), the first week of challenge (4d of challenge; CONT-1) and the fourth week of challenge (24 d of challenge; CONT-4). In each case, incubation in the rumen lasted for 24 and 48 h. Incubated grass hay contained 90.50% DM, 93.36% OM, 66.59% NDF, and 41.48% ADF (DM basis). Prior to in situ incubation the hay, derived from the same batch of forages as fed to cows, was cut into pieces of approximately 2 cm in length and weighed into nylon bags (20 cm × 10 cm, 150-μm pore size, Linker Industrie-Technik GmbH, Kassel, Germany) which were then sealed tightly with a cable binder and attached to a weighted chain to ensure the bags remain within the solid digesta of the rumen. Before incubation in the rumen, all bags were soaked in warm water (39°C) for 15 min to simulate saliva addition and account for the soluble content of the test hay [11]. Afterwards, the bags were inserted in the rumen and positioned in the ventral rumen sac [11]. At each incubation time point, the bags were removed from the rumen, rinsed, squeezed, and the residue was immediately transferred into cryo-tubes (Sarstedt, Nürnbrecht, Germany) and stored at -80°C for DNA extraction.

### Bacterial DNA extraction and sequencing and quality control

To preserve anaerobic cultures, in situ hay residues were thawed on ice until they became pliant, were cut into small pieces, and a 250-mg homogenized subsample was used for genomic DNA isolation using the PowerSoil DNA isolation kit (MOBIO Laboratories Inc., Carlsbad, CA). Samples were mixed with sodium dodecyl sulfate-containing buffer C1 and heated at 70°C for 10 min to ensure proper lysis of bacteria [12]. Samples were then bead-beaten to dissociate microbes from feed particles and to disrupt the bacterial cells [13]. This was followed by chemical removal of cell debris and PCR inhibitors and column-based isolation of total genomic DNA according to the manufacturer’s instructions. The isolated DNA concentration was determined by a Qubit 2.0 Fluorometer (Life Technologies, Carlsbad, CA, USA) using the Qubit dsDNA HS Assay Kit (Life Technologies). One 20μL aliquot of each sample for a total of 40 genomic DNA samples (Base-I n = 8, Base-C n = 8, INT n = 8, CONT-1 n = 8, CONT-4 n = 8) were sent for amplicon sequencing using a MiSeq Illumina sequencing platform and paired-end technology (Microsynth AG, Balach, Switzerland). Sequencing targeted the V3-V5 hypervariable region of the 16S rRNA gene using the primer set 357F (5′-CCTACGGGAGGCAGCAG-3′) and 926R (5′-CCGTCAATTCMTTTRAGT-3′) [7] to generate an approximate amplicon size of ~570 bp. 16SrRNA gene PCRs, library preparation and sequencing was performed by Microsynth. Libraries were constructed by ligating sequencing adapters and indices onto purified PCR products using the Nextera XT Sample Preparation Kit (Illumina) according to the recommendations of the manufacturer. Equimolar amounts of each of the libraries were pooled and submitted for sequencing on an Illumina MiSeq Personal Sequencer using a 300 bp read length paired-end protocol. After sequencing the corresponding overlapping paired-end reads were stitched by Microsynth. resulting in a total of 6,995,569 unfiltered reads with 517 ± 14 nt in length with a mean of 105,304 (SD ± 41786) sequences per sample.

Sequence quality control and analyses were performed using the QIIME pipeline [14]. Sequences were first quality filtered following previously published recommendations [15] and then screened for chimeras using the gold.fa database and USEARCH, filtered and then picked using UCLUST [16]. Finally, samples were aligned and clustered to define operational taxonominc units (OTUs) using PyNAST [14] and the Greengenes database as a reference template (version 13_8) [17]. The degree of similarity between sequences was defined as 97% to obtain OTU identity at the species level. Any OTUs which clustered with less than 10 reads were manually removed to ensure that unique OTUs are not over estimated. A total of 6,212,911 sequences clustered into 11,466 OTUs for diversity and PICRUSt analysis. Due to the large number of OTUs, all OTUs with a relative abundance greater than 0.1% (159 OTUs) were taken for statistical analysis. Samples were rarefied based on the minimum number of sequences found in a sample (37,845 sequences) prior to diversity analysis.

### Predicted functional domain analysis

Prediction of the genomic potential of the rumen bacterial sequences was performed with PICRUSt, based on 16S rRNA data (Phylogenetic Investigation of Communities by Reconstruction of Unobserved States) [18]. This tool is based on the assumption that phylogeny can reliably predict gene content. For PICRUSt, a closed reference OTU picking was performed with quality controlled sequences by aligning sequences to Greengenes reference OTUs (downloaded from http://greengenes.secondgenome.com/downloads/database/13_5) and OTUs were clustered based on 0.03 distance limit. This OTU table was used for PICRUSt on the online Galaxy interface (http://galaxyproject.org/), with a workflow described by the developers using the Kyoto Encyclopedia of Genes and Genomes (KEGG).

### Statistical analysis

Data of OTU and KEGG pathway relative abundances were analyzed with the 5 treatments (Base-I, INT, Base-C, CONT-1 and CONT-4) treated as independent measurements using the PROC MIXED of SAS (version 9.2; SAS Inst. Inc., Cary, NC) and a first-order autoregressive variance–covariance matrix was used to account for the repeated measures of individual animals [19]. The statistical model included main effects of treatment, incubation time and the interaction of these effects. Orthogonal contrasts were used to examine differences between challenge models, and baseline vs. SARA diet. Principle coordinate analysis (PCoA) was done using the UniFrac weighted metric and permutational multivariate analysis of variance (PERMANOVA) was implemented to test differences in beta-diversity among dietary phases and diet composition. The correlation matrix was used to generate principal component eigenvalues because variables were measured in different units. The loading plots of the first three components were considered. Degrees of freedom were estimated using the method of Kenward-Roger. Significance was indicated with superscripts for *P* ≤ 0.05 and trends were defined at a level of 0.05 < *P* ≤ 0.10. All values are reported as least squares means with standard error of the mean. Sequencing data are submitted in BioProjectSRA data base accession numbers SAMN06293117 to SAMN06293176.

## Results

Dietary composition, DMI, ruminal pH profiles and in situ nutrient degradation for the complete trial were previously reported [9, 20]. For this study, a subset of data from the microbial sampling days was used to provide correlation data (Table 1). Statistical analysis of DMI, mean pH and time spend under pH 5.8 (min) all showed a significant effect of treatment (*P* ≤ 0.01). Dry matter intake increased with the decrease in forage content fed in the high grain treatments (INT, CONT-1, CONT-4; *P* < 0.001). Mean rumen pH decreased correspondingly with decreased forage in the diet. The lowest pH (*P* < 0.001) and greatest time spent below pH 5.8 (*P* = 0.01) was determined for the INT sampling (pH = 5.96; time below pH 5.8 = 425 min) (Table 1).

**Table 1:** Dry matter intake (DMI) and pH taken on treatment sampling day^1^.

Parameter	Intermittent Feeding		Continous Feeding			SEM	*P*-value
	Base-I^2^	INT	Base-C	CONT-1	CONT-4		
DMI (kg per day)	10.9^b^	17.3^a^	9.9^b^	15.3^a^	17.3^a^	0.98	< 0.001
Mean pH	6.53^a^	5.96^b^	6.48	6.2	6.24	0.075	< 0.001
Time spent pH < 5.8 (min)	0^b^	425^a^	0	145	231	91.1	0.013

Values are least squares means ± standard error of the mean (SEM).

^**1**^Values are a mean of data from 24 and 48 h sampling points under two subacute ruminal acidosis (SARA) challenge models.

^a,b^ For each variable, means in the same feeding model within the same row differ, superscripts are based on Tukey's HSD test.

^2^Base-C = forage only diet for continuous challenge model, Base-I = forage only diet for intermittent challenge model, INT = SARA inducing diet for intermittent challenge model, CONT-1 = SARA inducing diet sample after one week of continuous challenge model, CONT-4 = SARA inducing diet sample after 4 weeks of continuous challenge model.

### Microbial diversity and phylogenetic analysis

The estimated measures of richness and diversity for rumen fiber associated microbes between forage-only and high grain diets in two SARA challenge models, as well as at 24h and 48h of ruminal incubation are shown in Table 2. While time of incubation in this experiment showed no effect on alpha diversity and richness, the total number of identified OTUs, as well as Chao, Simpson and Shannon indices all showed a significant effect of treatment (*P* ≤ 0.002; Table 2). For all measures, diversity and richness of the forage-associated microbes was higher in the forage based diets (Base-C and Base-I). Simpson indices showed the lowest levels of species diversity in the CONT-4 treatment (0.96) in comparison to forage based dietary treatments (*P* = 0.002).

**Table 2:** Alpha diversity measures of rumen fiber-associated communities based on treatment and duration of incubation.

Item	Intermittent Feeding		Continuous Feeding			SEM	Insitu Incubation (hours)		SEM	*P*-value	
	Base-I^1^	INT	Base-C	CONT-1	CONT-4		24h	48h		Phase^3^	Time
Observed OTUs^2^	2762^a^	1856^b^	2836^a^	1850^b^	1577^b^	97	2174	2178	61.3	< 0.001	0.97
Chao	3868^a^	2903^b^	4007^a^	2815^b^	2374^b^	141.9	3207	3180	89.7	< 0.001	0.84
Simpson	0.99^a^	0.97^b^	0.99^a^	0.97^a^^b^	0.96^b^	0.007	0.97	0.98	0.004	0.002	0.4
Shannon	9.00^a^	7.47^b^	9.11^a^	7.30^b^	6.85^b^	0.239	7.85	8.04	0.151	< 0.001	0.4

Values are least squares means ± standard error of the mean (SEM).

^a,b^ For each variable, means in the same feeding model within the same row differ, superscripts are based on Tukey's HSD test.

^1^Base-C = forage only diet for continuous challenge model, Base-I = forage only diet for intermittent challenge model, INT = SARA inducing diet for intermittent challenge model, CONT-1 = SARA inducing diet sample after one week of continuous challenge model, CONT-4 = SARA inducing diet sample after 4 weeks of continuous challenge model.

^2^ OTU = operational taxonomic unit to 97% identity

^3^ Phase = sampling point for either model

Based on percent abundance, OTUs were sorted according to relative contribution to the total ruminal sequences found in this study. A total of 159 OTUs were statistically analyzed each representing >0.1% relative abundance within the sequenced community with a total of 65.93% of the total sequences being accounted for. From the total OTU population, 49 OTUs had relative abundances greater than 0.5% of the rumen community in any of the samples, 22 of which showed a significant effect of treatment (Table 3). These OTUs were then secondary classified to the National Center for Biotechnology Information (NCBI) nucleotide database using Blastn for taxonomic classification and percent similarity with limitation to the 16S rRNA target (Table 3) [21]. The secondary classification was able to identify 4 OTUs to the species level with a percent similarity of 97% or higher, in addition to the 4 species already identified by Greengenes. Furthermore, blasting to the NCBI database was able to classify 5 OTUs to the genera level with greater than 94% similarity, including members of the genera *Sporobacter* (OTU 38 and 47) and *Flintibacter* (OTU 25 and 27; Table 1). *Sporobacter* is a gram positive, obligate anaerobe identified from the gut microbiome of termites [22]. *Flintibacter butyricus* is a novel gut microbe recently identified as a major butyrate producer which ferments amino acids glutamine and glutamate [23]. The lowest percent similarity for classification was 84% for both OTU 46 (*Endomicrobia*) and OTU 23 (*RFN20*). '*Endomicrobia*', is a distinct and diverse group of uncultivated bacteria represented so far only by intracellular symbionts of termite gut flagellates. Zheng et al. [24] reported the isolation and characterization of the first free-living member of this clade from sterile-filtered gut homogenate of defaunated (starch-fed) termites. The genus *RFN20* is a rumen specific genus from the *Tenericutes* phylum [25].

**Table 3:** Percent relative abundance and taxonomic classification of significant^1^ OTUs representing greater than 0.5% ruminal abundance.

OTU^2^	Feeding Phases^3^					SEM	*P*-value	NCBI^4^ Classification	Percent identity to NCBI^5^,^6^
	Intermittent Feeding		Continous Feeding						
	Base-I	INT	Base-C	CONT-1	CONT-4				
OTU1	5.75^a^	2.66^b^	5.11	4.34	3.01	0.611	0.005	*Saccharofermentans acetigenes*	98
OTU3	0.12	1.66	0.13	3.52	7.36	1.745	0.03	*Ruminobacter amylophilus*	86
OTU5	0.81^b^	2.74^a^	0.46^b^	2.40^a^	1.00^a^^b^	0.508	0.01	*Selenomonas ruminantium*	99
OTU6	0.79^b^	2.48^a^	0.46^b^	1.64^a^^b^	1.89^a^	0.389	0.01	*Succiniclasticum ruminis*	100
OTU7	0.90^b^	1.82^a^	1.11	1.25	2.5	0.354	0.02	*Ruminococcus flavefaciens*	99
OTU10	1.84^a^	1.06^b^	1.97^a^	1.85^a^^b^	0.82^b^	0.26	0.01	*Alkalibaculum* genus	88
OTU11	1.00^b^	1.54^a^	0.83^b^	1.49^a^^b^	1.70^b^	0.179	0.01	*Succiniclasticum ruminis*	99
OTU12	1.83^a^	0.72^b^	1.92^a^	1.65^a^	0.52^b^	0.169	<0.0001	*Gracilibacter genus*	86
OTU21	0.42^b^	1.11^a^	0.26	0.91	0.93	0.188	0.01	*Succiniclasticum ruminis*	95
OTU23	0.32^b^	0.84^a^	0.41	0.42	1.35	0.254	0.04	*Holdemania genus*	84
OTU24	0.65	1.03	0.59^a^^b^	0.92^a^	0.45^b^	0.119	0.01	*Intestinimonas* genus	93
OTU25	0.84	0.83	0.82^a^	0.83^a^	0.40^b^	0.093	0.01	*Flintibacter butyricus*	94
OTU27	1.04^a^	0.55^b^	0.89	0.6	0.53	0.102	0.003	*Flintibacter butyricus*	94
OTU32	0.52	0.56	0.46	0.46	1.04	0.148	0.05	*Intestinimonas* genus	93
OTU33	0.17^b^	1.14^a^	0.07	0.66	0.6	0.205	0.01	*Anaerovibrio lipolyticus*	99
OTU34	0.96^a^	0.32^b^	1.08^a^	0.52^b^	0.18^b^	0.12	<0.0001	*Gracilibacter genus* genus	88
OTU35	1.10^a^	0.19^b^	1.02^a^	0.47^b^	0.17^b^	0.126	<0.0001	*Ruminococcus flavefaciens*	98
OTU38	0.99^a^	0.09^b^	1.03^a^	0.48^a^^b^	0.35^b^	0.14	<0.0001	*Sporobacter termitidis*	94
OTU40	1.17^a^	0.28^b^	0.76^a^	0.14^b^	0.03^b^	0.161	<0.0001	*Sutterella stercoricanis*	92
OTU46	0.52^a^	0.18^b^	0.84^a^	0.24^b^	0.23^b^	0.102	<0.0001	*Elusimicrobium* genus	84
OTU47	0.73^a^	0.05^b^	0.77^a^	0.38^a^^b^	0.19^b^	0.097	<0.0001	*Sporobacter termitidis*	94
OTU49	0.53^a^	0.14^b^	0.66	0.12	0.5	0.125	0.01	*Ruminococcus flavefaciens*	99

Values are least squares means ± standard error of the mean (SEM)

^1^All OTUs presented in this table have a significant effect of treatment (*P* ≤ 0.05)

^2^OTU = operational taxonomic unit to 97% identity, numbered based on highest percent abundance and continuing in decreasing order

^3^Base-C = forage only diet for continuous challenge model, Base-I = forage only diet for intermittent challenge model, INT = SARA inducing diet for intermittent challenge model, CONT-1 = SARA inducing diet sample after one week of continuous challenge model, CONT-4 = SARA inducing diet sample after 4 weeks of continuous challenge model.

^4^Contrasts refers to orthogonal contrast analysis

^5^NCBI = National Center for Biotechnology Information

^6^Percent identity (%)

^a,b^ For each variable, means in the same feeding model within the same row differ, superscripts are based on Tukey's HSD test.

Of the 22 fiber-adherent OTUs significantly affected by treatment (Table 3), 16 were significantly different between the Base and the high grain treatments according to contrast analysis of individual OTUs. The OTU 3, classified as belonging to the *Succinivibrionaceae* family was only found to be significantly impacted by CONT-4 with an increase to more than 7% relative abundance. The interrupted feeding model significantly impacted OTU 5 (*Selenomonas ruminantium*) and OTU 24 (*Ruminococcaceae* family) with the highest percent relative abundance during the INT treatment in comparison to the Base-I (Table 3).

**Fig 1:**
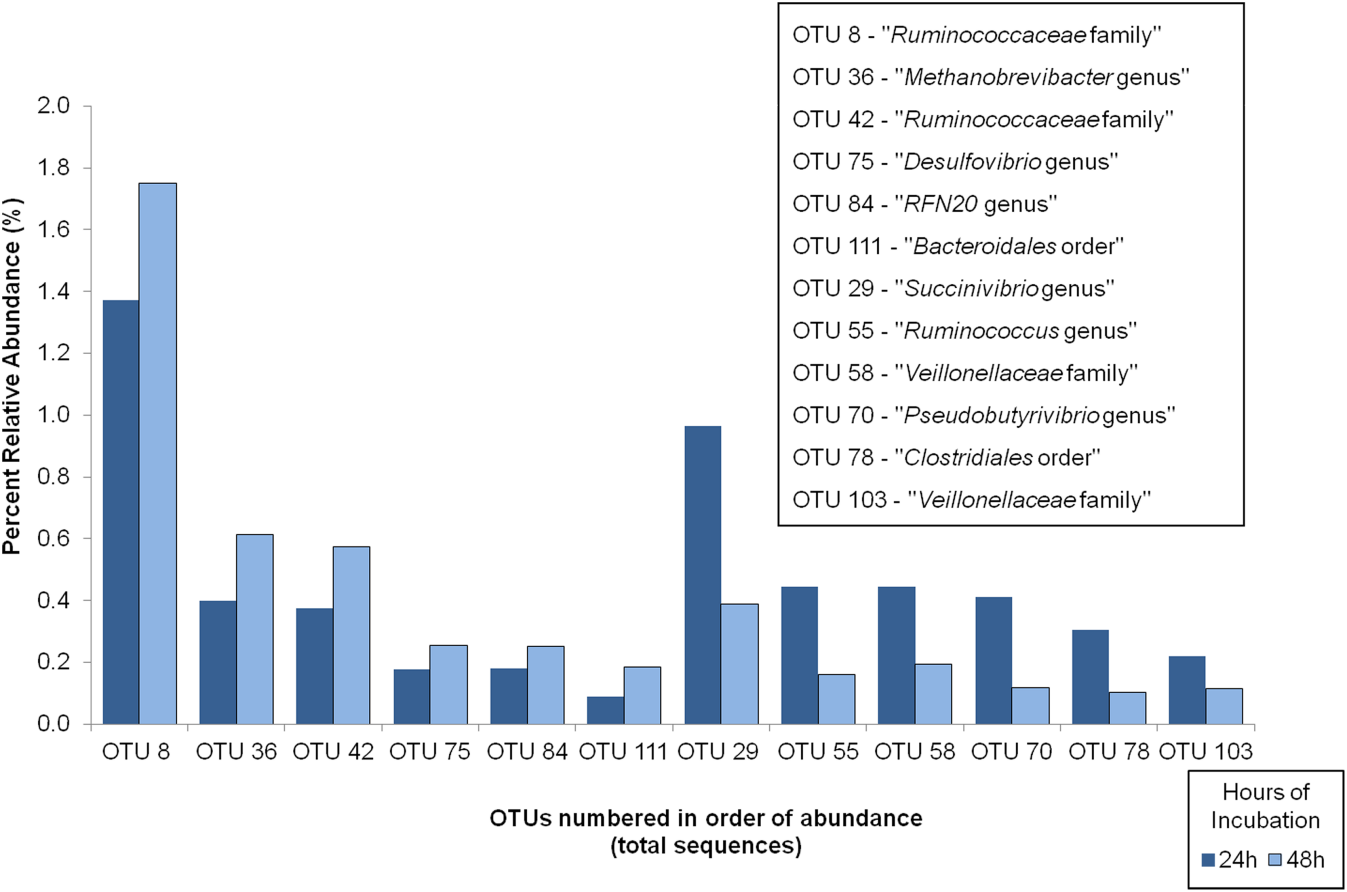
Mean values of OTUs combined with significant variation between continuous and interrupted challenge models.

Statistical analysis using orthogonal contrast analysis comparing the 159 OTUs (representing 0.1% of the total sequences), found 7 OTUs which showed a significant effect or trend towards significance of challenge model based on percent relative abundance (Fig 1). Those with a significant effect of contrasted challenge models were OTU 118 (Family *BS11*; *P* = 0.03) and OTU 119 (*Ruminococcus* genus; *P* = 0.01).

**Fig 2:**
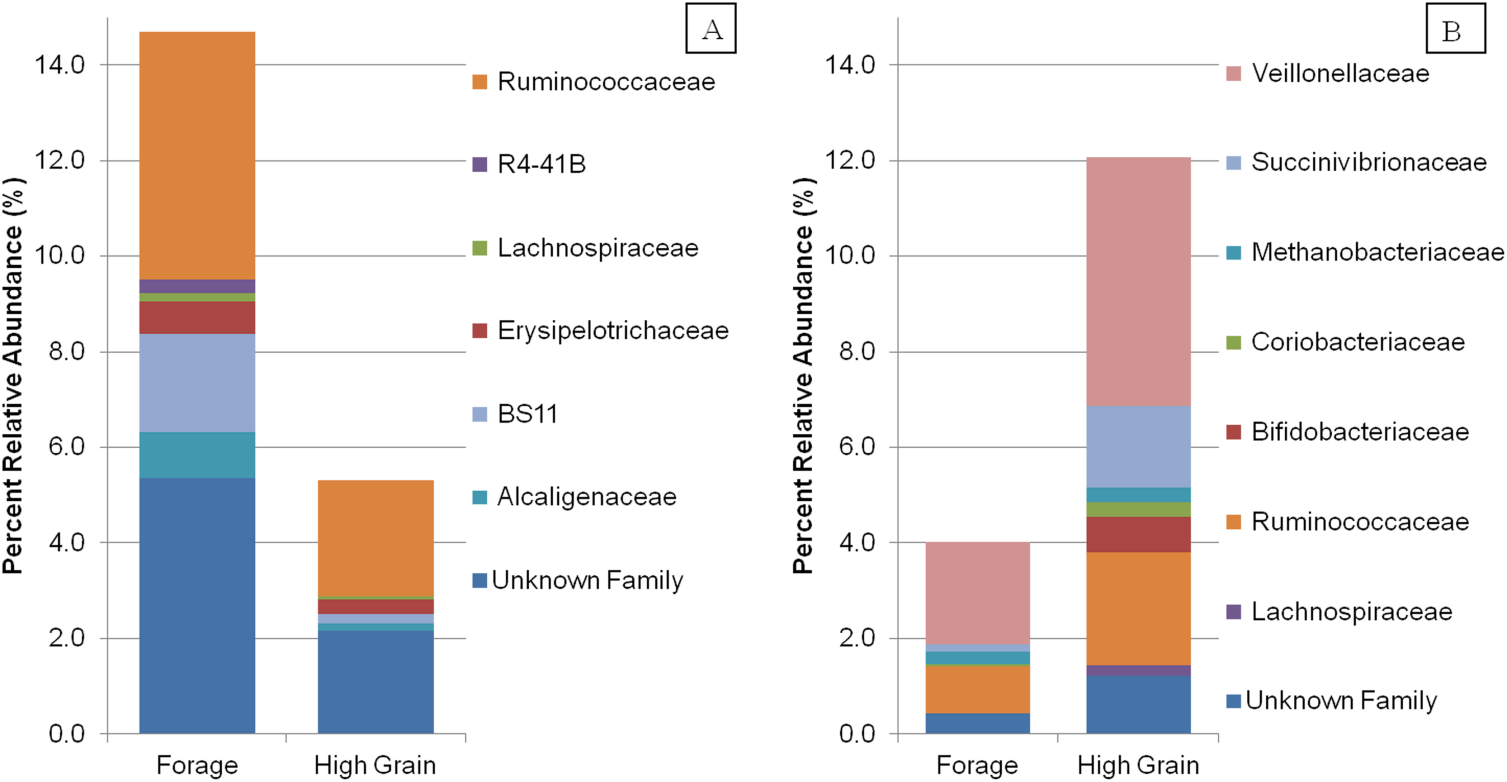
Principal coordinate analysis of the beta-diversity of rumen in-situ samples using weighted UniFrac. Analysis by PERMANOVA revealed a diet effect (P = 0.001) and an effect of feeding phase (P = 0.03).

**Fig 3:**
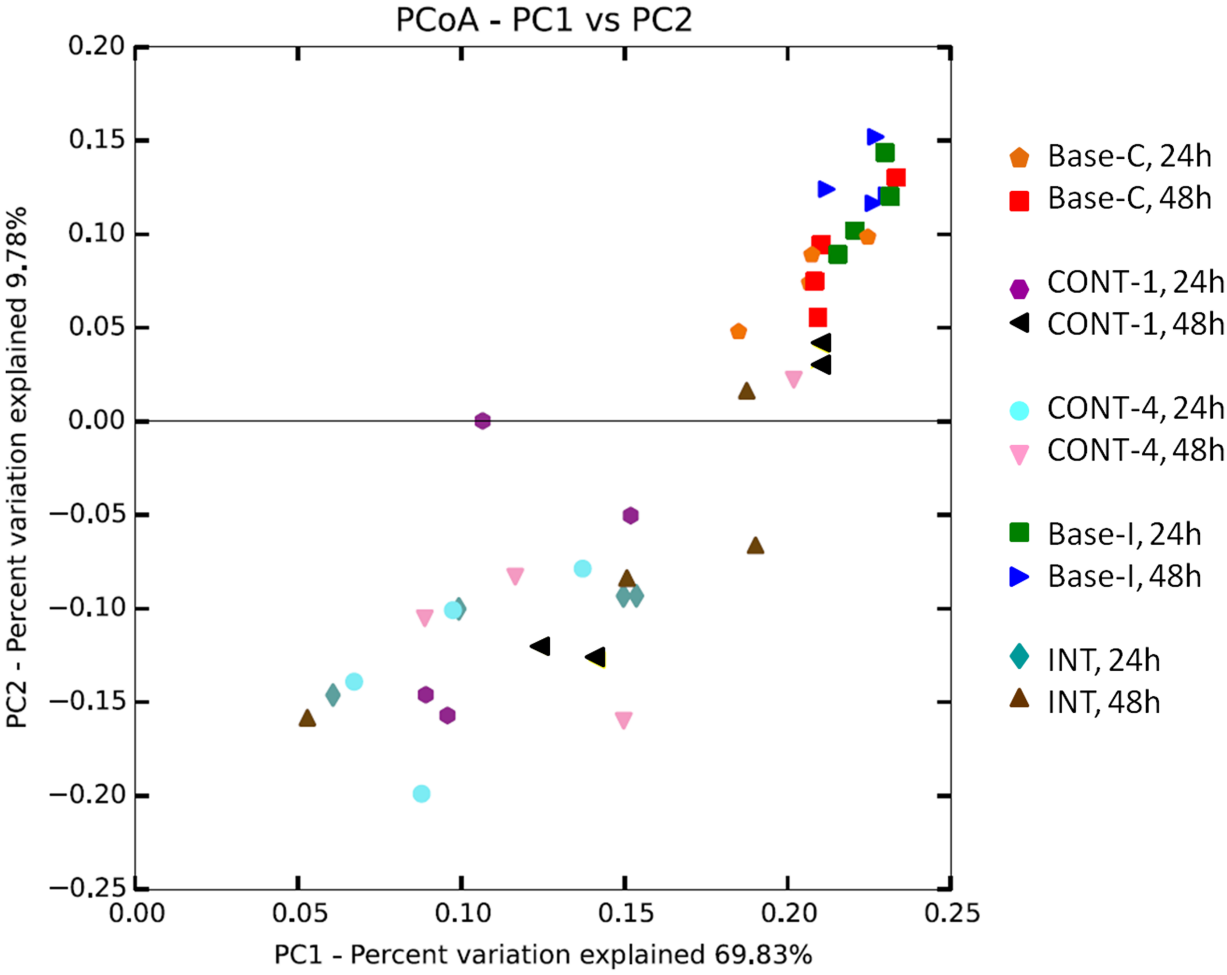
Mean percent relative abundance of family groups between diets. (A) Decreasing percent abundance (B) Increasing percent abundance in the SARA diet in comparison to the forage diet.

Using principal coordinate analysis of fiber-adherent microbiota indicated a clear clustering of microbial communities between baseline and grain diet whereby (Fig 2). Based on family taxons, significant OTUs were sorted based an overall increase (Fig 3A) or decrease (Fig 3B) in percent relative abundance between the forage and grain feeding. Even at the taxonomic classification of family, a large portion of OTUs could not be classified. Several families were shown to decrease significantly in grain feeding including *Erysipelotrichaceae*, *BS11*, *Alcaligenaceae* and *R4-41B*, compared with forage feeding. Familes *Ruminococcaeceae*, *Lachnospiraceae* and those classified as "unknown" at the Family level had members who both either increased in the grain diet or decreased in the grain diet compared with forage feeding. Families *Veillonellaceae*, *Succinivibrionaceae*, *Coriobacteriaceae*, *Bifidobacteriaceae* and the Archaea family *Methanobacteriaceae* all increased significantly with the change to a grain diet.

**Fig 4:**
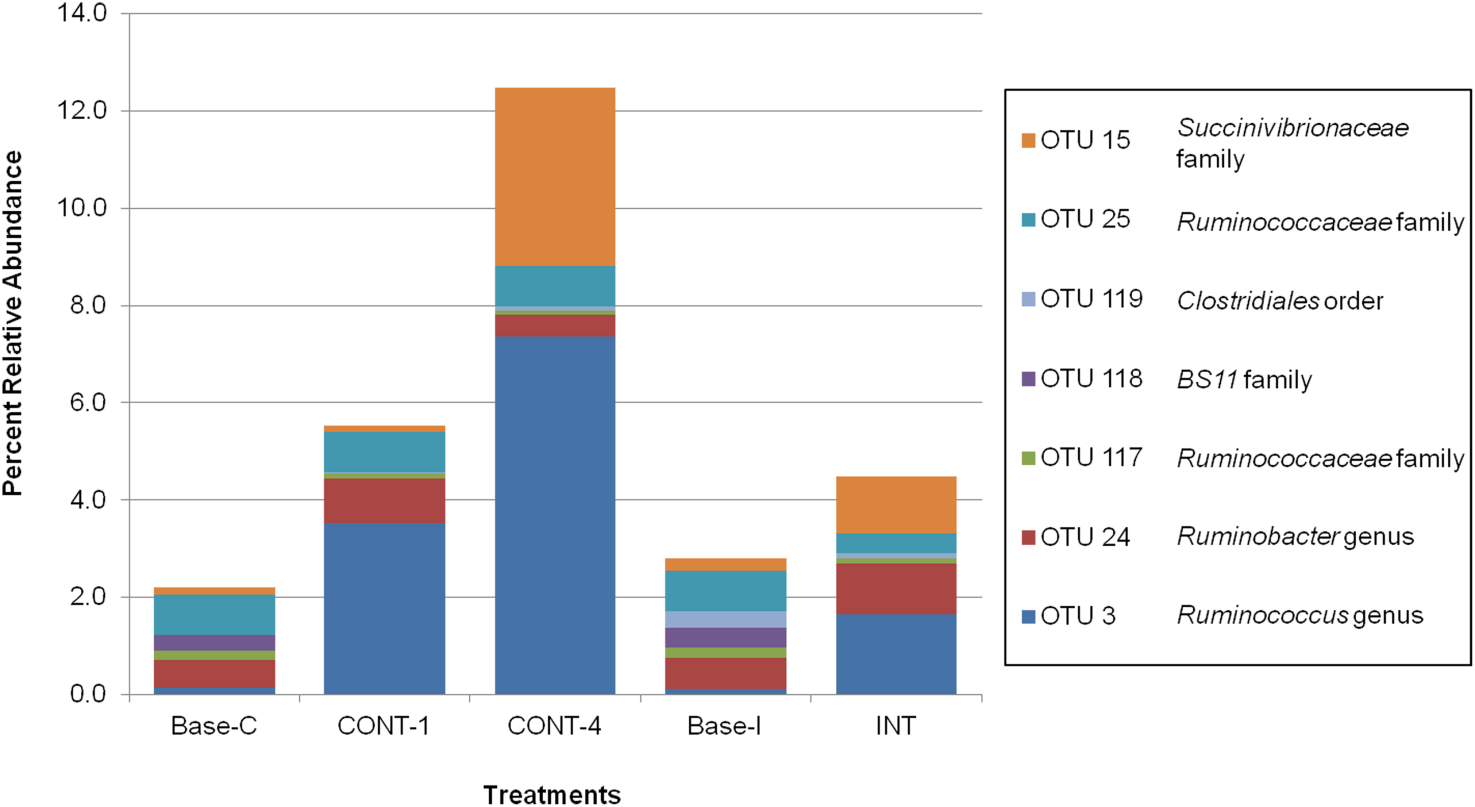
Effect of in situ incubation time on OTUs classified by the GreenGenes database.

Of the 159 hay-associated OTUs analyzed, 12 were significantly impacted by incubation time (Fig 4). These OTUs classified predominantly at the genus or family taxonomic level with the exception of OTU 111 (order *Bacteroidales*) which increased from 24 to 48 h of incubation and OTU 78 (order *Clostridiales*) which decreased from 24 to 48h of incubation. Sequence analysis of 0-h in situ bags, soaked in water but not ruminally incubated, showed a bacterial community predominated by *Proteobacteria* and therefore, data were not normalized for the time 0 h population.

Pearson correlation of OTUs representing a percent relative abundance of 0.5% or greater were analyzed with relation to the environmental parameters DMI, mean pH and the duration of time spend under pH 5.8, as a reference point for environmental effects on rumen cellulolytic populations [26]. From the 49 OTUs that were analyzed, 9 showed highly significant (*P* ≤ 0.001) correlations to one or more of the parameters (Table 4). Correlations shown are those which had a Pearson correlation coefficient of greater than 0.50. Two OTUs correlated to all three environmental parameters, OTU 38 and OTU 47, in a similar pattern with a negative correlation to DMI and duration of time below pH 5.8 and a positive correlation to mean pH. Both these OTUs were taxonomically classified as belonging to the family *Ruminococcaceae*. The highest correlation determined (r = -0.73) was for the OTU 21, which identified to 97% with *Succiniclasticum spp*., with mean pH, indicating that as mean pH decreased, the relative abundance of this OTU increased. This was also found for OTU 6 which was also identified as genus *Succiniclasticium*. Correlation analysis of rumen environmental parameters and microbial diversity indices showed no significant relations.

**Table 4:** Pearson correlation coefficients for OTUs^1^ with rumen environmental parameters.

OTU^2^ ranking	Greengenes taxonomic classification (97%)	DMI^3^	Mean pH	Duration of time (min) spend under pH 5.8
6	*Succiniclasticum* genus	ns	-0.63	0.50
12	*Clostridiales* order	0.60	ns	ns
21	*Succiniclasticum* genus	ns	-0.73	0.64
33	*Anaerovibrio* genus	ns	-0.50	ns
34	*Clostridiales* order	ns	0.55	ns
35	*Ruminococcus flavefaciens*	ns	0.61	-0.50
38	*Ruminococcaceae* family	-0.57	0.61	-0.53
46	*Endomicrobia* class	-0.56	0.54	ns
47	*Ruminococcaceae* family	-0.61	0.63	-0.54

ns: not significant

^1^Analysis was performed for OTUs (≥0.5% of the relative abundance) which showed a significant effect of treatment. All OTUs shown in this table had a P< 0.001 for the given correlations.

^2^OTU = operational taxonomic unit to 97% identity, ranked in decreasing order based on total percent abundance

^3^DMI = dry matter intake (kg per day)

### Predicted functional pathway analysis of fiber-adherent microbes

A total of 66 predicted functional pathways, representing more than 0.5% percent abundance were statistically analyzed from the 16S rRNA quality controlled sequences (Table 5). The 28 functional pathways, found to be significantly affected by treatment, grouped under 4 categories of cellular processes (1.11% abundance), environmental information processing (0.74%), genetic information processing (7.97%) and metabolism (20.59%) representing an average relative abundance of 30.4% of the predicted function based on analyzed microbial sequences. From the 28 pathways, 4 pathways had an additional effect of incubation time and one pathway showed a treatment by time interaction (Table 5). Predicted functional pathways affected by incubation time included those under the categories of genetic information processing and metabolism. Changes in predicted functionality of the fiber-adherent rumen microorganisms included an increase in predicted methane metabolism pathways between 24 and 48h of incubation, and a decrease in predicted glyoxylate metabolism pathway over incubation time (Table 5). The treatment by time interaction was noted for the metabolism of amino sugar and nucleotide sugar with the a notable increase in the relative abundance of this predicted functional pathway in the high concentrate diets compared to the forage diets. Analysis of the interaction using mean comparison tests indicated that the Base-C at 24h was significantly different (*P* ≤ 0.05) from the CONT-4 at 24h as well as between the CONT-4 at 24h and the INT at 24h (data not shown).

**Table 5:** Predicted functional pathway analysis using the Kyoto Encyclepedia of Genes and Genomes (KEGG).

KEGG Pathway ID	Intermittent Feeding		Continous Feeding			SEM	Insitu Incubation (hours)		SEM	*P*-value		
	Base-I^1^	INT	Base-C	CONT-1	CONT-4		24h	48h		Phase	Time	Phase × Time
**Cellular Processes**												
Cell cycle	0.63	0.61	0.63	0.61	0.63	0.005	0.62	0.62	0.003	0.004	0.37	0.24
Cytoskeleton proteins	0.47^b^	0.51^a^	0.47	0.5	0.48	0.009	0.49	0.48	0.006	0.01	0.8	0.36
**Environmental Information Processing**												
Bacterial secretion system	0.69^b^	0.76^a^	0.69^b^	0.77^a^	0.76^a^	0.016	0.75	0.72	0.01	0.001	0.06	0.46
**Genetic Information Processing**												
Chromosome	1.93^b^	1.99^a^	1.93^b^	2.02^a^	2.03^a^	0.015	2	1.96	0.01	< 0.001	0.01	0.87
Transcription factors	1.84	1.92	1.81^a^^b^	1.93^a^	1.77^b^	0.038	1.84	1.87	0.024	0.03	0.32	0.2
DNA replication proteins	1.61^a^	1.53^b^	1.63	1.58	1.57	0.018	1.58	1.59	0.012	0.007	0.55	0.91
Transcription machinery	1.39^a^	1.21^b^	1.40^a^	1.28^a^	1.26^b^	0.03	1.28	1.34	0.019	< 0.001	0.04	0.53
Translation factors	0.71^a^	0.69^b^	0.72	0.69	0.7	0.006	0.7	0.71	0.004	0.01	0.15	0.95
Base excision repair	0.53^b^	0.55^a^	0.53	0.55	0.55	0.005	0.54	0.54	0.003	0.01	0.32	0.82
**Metabolism**												
Pyrimidine metabolism	2.33	2.29	2.35^a^	2.30^a^^b^	2.28^b^	0.017	2.3	2.32	0.011	0.02	0.12	0.87
Amino acid related enzymes	1.87	1.85	1.88^a^^b^	1.84^b^	1.88^a^	0.009	1.87	1.86	0.006	0.01	0.49	0.09
Methane metabolism	1.65	1.63	1.67^a^	1.63^a^^b^	1.54^b^	0.028	1.59	1.66	0.018	0.04	0.01	0.31
Amino sugar and nucleotide sugar metabolism	1.57	1.59	1.57^b^	1.60^a^^b^	1.63^a^	0.01	1.59	1.59	0.006	0.002	0.95	0.03
Arginine and proline metabolism	1.46	1.47	1.46	1.45	1.42	0.011	1.44	1.46	0.007	0.05	0.14	0.48
Oxidative phosphorylation	1.37^a^	1.24^b^	1.38^a^	1.29^b^	1.37^a^^b^	0.025	1.33	1.33	0.016	0.001	0.73	0.54
Carbon fixation pathways in prokaryotes	1.29^a^	1.21^b^	1.30^a^	1.24^b^	1.23^b^	0.016	1.24	1.27	0.01	< 0.001	0.09	0.84
Phenylalanine, tyrosine and tryptophan biosynthesis	1.10^b^	1.17^a^	1.10^b^	1.13^a^^b^	1.16^a^	0.011	1.14	1.12	0.007	< 0.001	0.13	0.75
Fructose and mannose metabolism	0.91^b^	1.00^a^	0.92^b^	0.99^a^	0.96^a^^b^	0.017	0.96	0.95	0.011	0.002	0.65	0.62
Pentose phosphate pathway	0.89^b^	0.99^a^	0.90^b^	0.98^a^	0.92^a^^b^	0.021	0.94	0.93	0.013	0.005	0.57	0.52
Pantothenate and CoA biosynthesis	0.82^a^	0.78^b^	0.82^a^	0.78^b^	0.81^a^^b^	0.008	0.8	0.81	0.005	0.001	0.23	0.55
Nitrogen metabolism	0.83^a^	0.78^b^	0.82	0.77	0.78	0.015	0.79	0.81	0.01	0.02	0.22	0.8
One carbon pool by folate	0.82^a^	0.76^b^	0.82^a^	0.77^b^	0.80^a^^b^	0.01	0.79	0.8	0.006	< 0.001	0.35	0.74
Galactose metabolism	0.69^b^	0.74^a^	0.68	0.71	0.69	0.014	0.7	0.7	0.009	0.04	0.97	0.58
Glycerophospholipid metabolism	0.67^a^	0.65^b^	0.68	0.66	0.67	0.007	0.67	0.67	0.004	0.03	0.84	0.27
Propanoate metabolism	0.68^a^	0.66^b^	0.69^a^	0.65^b^	0.65^b^	0.008	0.66	0.67	0.005	0.003	0.47	0.51
Thiamine metabolism	0.66	0.64	0.65^b^	0.65^b^	0.68^a^	0.006	0.65	0.65	0.004	0.002	0.86	0.49
Glyoxylate and dicarboxylate metabolism	0.52^b^	0.59^a^	0.52^b^	0.57^a^^b^	0.58^a^	0.012	0.57	0.54	0.007	< 0.001	0.02	0.67
Pentose and glucuronate interconversions	0.47^b^	0.55^a^	0.48	0.53	0.48	0.016	0.51	0.49	0.01	0.008	0.31	0.61

Values are least squares means ± standard error of the mean (SEM).

^a,b^ For each variable, means in the same feeding model within the same row differ, superscripts are based on Tukey's HSD test.

^1^Base-C = forage only diet for continuous challenge model, Base-I = forage only diet for intermittent challenge model, INT = SARA inducing diet for intermittent challenge model, CONT-1 = SARA inducing diet sample after one week of continuous challenge model, CONT-4 = SARA inducing diet sample after 4 weeks of continuous challenge model.

### Correlation of predicted pathways with rumen environmental parameters

Predicted metabolism functional pathways which showed a significant impact of treatment, time of incubation or the interaction thereof were further analyzed for Pearson correlation coefficients (r) to rumen environmental parameters of mean pH, duration of time (min) spent under pH 5.8 as a marker of SARA and the average DMI (kg/day; Table 6). This analysis produced 6 predicted pathways with a significant correlation to one or more of the environmental parameters with a r value of greater than 0.5 or less than -0.5 (Table 6). All predicted functional metabolism pathways were significantly correlated to mean pH, only one of which, glyoxylate metabolism, was a negative correlation. Oxidative phosphorylation was the only pathway not significantly correlated to the duration of time (min) spent under the pH 5.8 and DMI, whereas the metabolism of carbon by folate was not significantly impacted by DMI. Except for glyoxlate metabolism, all other pathways showed a negative correlation to duration under pH 5.8 and DMI (kg/day).

**Table 6:** Correlation analysis of significant predicted functional functional pathways (KEGG^1^) to rumen environmental parameters.

KEGG Predicted Functional Pathway		Pearson Correlation Coefficient (r) / *P*-Value of Correlation	Mean pH	Duration (min) under pH 5.8	DMI^2^
Metabolism					
Energy Metabolism	Carbon fixation pathways in prokaryotes	r	0.80	-0.72	-0.42
		*P*-value	< .0001	< .0001	0.01
Energy Metabolism	Oxidative phosphorylation	r	0.50	-0.28	-0.20
Metabolism of Cofactors and Vitamins		*P*-value	0.001	0.08	0.21
	One carbon pool by folate	r	0.60	-0.41	-0.30
		*P*-value	< .0001	0.01	0.06
Carbohydrate Metabolism	Propanoate metabolism	r	0.53	-0.45	-0.38
		*P*-value	0.0004	0.004	0.01
Carbohydrate Metabolism	Glyoxylate and dicarboxylate metabolism	r	-0.61	0.49	0.43
		*P*-value	< .0001	0.001	0.01
Nucleotide Metabolism	Pyrimidine metabolism	r	0.65	-0.67	-0.43
		*P*-value	< .0001	< .0001	0.01

Values are least squares means ± standard error of the mean (SEM).

^1^ Kyoto Encyclepedia of Genes and Genomes

^2^ DMI = dry matter intake (kg per day)

## Discussion

Previous research looking at the impact of feeding models on nutrient degradation indicated that there were changes in fiber degradation unrelated to pH (Pourazad et al. 2017). Based on this, these samples were used for microbial analysis to determine the impact of feeding model on the fiber-adherent in-situ microbial population. Since the vast majority of rumen microbial research associated with high grain feeding and SARA assume that animals undergo an extended period of SARA [4, 27]. However, intermittent bouts of SARA and interrupted periods of high grain feeding may more closely represent episodes of SARA on farms under high grain feeding conditions [7]. In the present study, we elucidated the effect of an interrupted high grain feeding model on the in situ fiber-adherent rumen microbial population and its adaptation to the environmental parameters such as DMI and pH, as well as the impact of short term (24 to 48 h) incubation on the rumen bacterial community diversity, phylogeny and predicted functional metabolic pathways. As noted by Pourazad et al. [9], pH data showed that SARA was more severe in the INT model than in the CONT model. This difference was unaccounted for by variation in DMI and grain intake [9]. This data verified that the feeding models used in this experiment were in fact significantly different in regards to the severity of a SARA challenge.

### Changes in the in situ fiber-adherent microbiota based on challenge model and incubation length

For all measures of richness and diversity, the CONT-4 samples showed the greatest decrease in comparison to samples taken in the baseline for either model. Our hypothesis was that the interruption in a SARA challenge would create an unstable environment; however, pH data showed that the interrupted challenge model showed the most severe challenge but CONT-4 had the lowest microbial diversity. While both models showed clear differences between the SARA and forage diets, it is unclear whether the differences are based on rumen instability caused by dietary changes (INT) or whether it is based on the length of the SARA challenge (CONT-4).

Analysis of percent abundance of OTUs based on treatment (Table 3) indicated that several OTUs were found at levels significantly higher in the CONT-4 samples compared to other treatments including *Ruminobacter amylophilus*, *Succiniclasticum ruminis*, *RFN20* genus, and *Intestimonas* genus. Potentially indicating a preference of these organisms to extended periods of low pH or high grain substrates. Conversely, increased levels of OTU 5 and 24 in the CONT-1 treatment as well as the INT treatment in comparison to the CONT-4 treatment indicates a potential opportunistic metabolism of *Selenomonas ruminantium* and *Intestimonas* spp. that allow for increased population growth under conditions of rumen microbial instability such as those seen during and shortly after dietary adaptation. Similar to the current study, Petri et al. [27] also reported increased abundance of *Selenomonas* spp. under low pH conditions, however, increases in *Intestimonas* spp. were not found. Analysis of OTUs based on the challenge model (Fig 1) showed fewer OTUs which were significantly impacted by the intermittent versus continuous model of SARA.

Previous research has looked at the impact of a transiently induced SARA challenge on the rumen epimural community using 16S rRNA sequencing [7]. However, in contrast to their results, which found decreasing levels of *Ruminobacter amylophilus* in the re-challenge period, the results of the current study agree with older work which found *R*. *amylophilus* to be amylolytic and abundant in high starch diets [28, 29]. Perhaps most interesting was that two OTUs, both identified as *Ruminococcus flavefaciens*, showed divergent responses to feeding models and sampling points (Fig 3). Despite our ability to identify ruminal bacteria to the species level, the variations within subspecies for substrate metabolism and environmental adaptation remains large. Furthermore, large variation found between individual animals make the impact of treatment and challenge model based on phylogenetic classification is therefore difficult to elucidate.

Statistical comparisons of the abundance of in situ fiber-adherent OTUs between 24 and 48h of incubation found 6 OTUs which significantly decreased in this time and 6 which significantly increased. The only archaea OTU found, genus *Methanobrevibacter*, significantly decreased percent abundance at 48 h compared to 24 h of in situ incubation (Figs 3 and 4). Piao et al. [25] also found *Methanobrevibacter* in the in situ fiber-adherent microbial community and also reported a decrease in abundance of *Methanobrevibacter* between 24 and 48h of incubation with in situ degradation of switchgrass. The majority of bacterial OTUs identified as having significant changes in percent abundance based on the duration of in situ degradation had also been previously reported as part of the fiber-adherent rumen microbiota [25]. However, of those genera noted both in the present study and by Piao et al. [25] only the OTU identified as *Pseudobutyrivibrio* spp. showed a decrease in relative abundance from 24 to 48 h of incubation in both studies. In the current study, *Clostridiales* and *Veillonellaceae* related OTUs were all noted as decreasing significantly in the 24 h between the two incubation sampling times. Piao et al. [25] found three *Veillonellaceae* related OTUs, two of which showed a decrease between 24 and 48h of incubation. In the present study, three separate OTUs were identified to the *Ruminococcaceae* family level, interestingly two of which increased over time and one of which decreased between 24 and 48h. Piao et al. [25] also found two OTUs within the *Ruminococcaceae* family identified to the genus' *Oscillospira* and *Ruminococcus* spp.; however, both of which showed increases over time. The comparability of the data from the present study with previous research from another group [1, 2] was unexpected due to variation in incubated substrate and dietary composition. Therefore, the similarities found would tend to indicate a highly conserved fiber-adherent microbial community within ruminants as part of the "core microbiome" as previously published [30].

Correlation of significant OTUs representing a relative abundance of ≥ 0.5%, to rumen environmental parameters of DMI, mean pH and the duration of time spend under pH 5.8 was performed to determine those fiber-adherent OTUs which were most likely impacted by changes to the in situ bag ecosystem (Table 4). The two OTUs which correlated to all three environmental parameters showed a negative correlation to DMI and the duration of time below pH 5.8 and a positive correlation to mean pH. Our results showed that the challenge model had no effect on these OTUs but that they were significantly higher in the forage diets compared to high grain. Both these OTUs were taxonomically classified as belonging to the family *Ruminococcaceae*, a group of predominantly cellulolytic bacteria [28], commonly found to be decreased under SARA conditions [1, 27]. Conversely to the cellulolytic bacteria, the highest negative correlation determined (r = -0.73) was for OTU 21 (*Succiniclasticum* genus) with mean pH, indicating that as mean pH decreased the relative abundance of this OTU increased. Myer et al. [29] found increased levels of *Succiniclasticum ruminis* in ruminant animals showing a lower efficiency of growth. While our study did not measure growth efficiency, our results show a clear effect of challenge model on OTU 21, with highest percent abundances found in the INT samples in comparison to CONT. In situ microbiota samples taken at the INT sampling represent the fiber-adherent microbial community after repeated dietary changes and could be representative of an unstable fiber-associated community. *Succiniclasticum ruminis* is a gram-negative bacteria with a very limited substrate profile, fermenting succinate to proprionate [31] however, its role in the rumen microbial ecosystem remains unclear.

### Predictive functional analysis

Phylogenetic investigation of communities by reconstruction of unobserved states (PICRUSt) is a computational approach to predict the functional composition of a metagenome using genomic data [18]. As expected, under variable environmental conditions and varied substrate availability, a number of predicted functional capacities of the in situ fiber-adherent rumen microbiome were significantly impacted by treatment group.

A total of 6 predicted metabolism related pathways were significantly correlated to mean pH. However, only one of which, glyoxylate metabolism, was a negative correlation. The glyoxylate cycle is an important subset of these reactions necessary to replenish the two-carbon precursors which enter the tricarboxylic acid cycle as acetyl-coenzyme A [32]. A decrease in the relative expression of this pathway as mean pH increases indicates a potential shift to this predicted function under low pH conditions. Since the equal and opposite correlation was seen in propanoate metabolism in relation to environmental parameters, this may indicate that the shift in carbohydrate metabolism under low pH for extended periods of time is at least partially shifted between these pathways. This was supported by the correlation analysis between the glyoxylate/dicarboxylate and propanoate metabolism pathways which showed a highly significant negative correlation (*P* = 0.0002; r = -0.55).

Previous research [32] suggested that control of the glyoxylate pathway is mediated through a negative feedback loop based on the intracellular concentrations of phosphoenolpyruvate, which is also the starting material for gluconeogenesis, the formation of cell-wall components and the synthesis of aromatic amino acids (i.e. histidine). Further correlation analysis indicated 10 OTUs which were highly correlated (0.5 ≥ r ≥ -0.5) to either or both pathways, all of which belonged to the order *Clostridiales* in either family *Ruminococcaceae* or *Veillonellaceae*. The four OTUs belonging to family *Veillonellaceae* all showed a positive correlation to glyoxylate/dicarboxylate metabolism and the three OTUs which were identified as *Succiniclasticum* spp. had negative correlations to propanoate metabolism. Since an increase of *Succiniclasticum* spp. on high grain diets is well documented [31] and in this study carbohydrate metabolism based on the glyoxylate pathway is increased in correlation with this species, this gram-negative microorganism may be a candidate for further analysis with relation to animals undergoing SARA.

Understanding the shifts in carbohydrate metabolism based on the rumen environment and microbial community could help to elucidate key compounds such as phosphoenolpyruvate for future SARA related metabolome research. However, it is important to pursue further research in this area with the use of additional molecular tools such as transcriptome analysis. The predictive nature of PICRUSt as a computational tool is limited by the accuracy and comprehensiveness of the database of reference genomes. Environments which are less extensively covered in the reference database, such as the rumen, are less accurately described by predictive analysis. While results obtained in this study provide indications of metabolic changes within the in situ rumen microbiome, the NSTI values of our study indicate low coverage (0.18±0.6). Therefore, PICRUSt usage alone within the rumen is insufficient to provide an accurate depiction of the predicted functional metagenome. However, the use of PICRUSt as an affordable approach prior to more expensive 'omics analysis is suitable due to the thousands of uncultivated microbial communities for which only marker gene surveys are currently available.

### Conclusion

The application of two challenge models (interrupted and continuous) showed an overall decrease in the diversity after 4wk of continuous SARA exposure compared to the interrupted model. Approximately 15% of individual OTUs identified were impacted by the challenge model. The majority of these OTUs correlated to DMI, mean ruminal pH and the duration of time spent under pH 5.8 as an indicator of SARA. Predictive functional pathway analysis provided indication that *Succiniclasticum* spp. may be positively impacted by the acidic conditions in the rumen under SARA and therefore a target for future SARA research. Changes in the fiber-adherent in situ community and their predicted metabolic function between 24 h and 48 h of incubation were also shown, however, difficulties remain in interpretation of these results with reference to the rumen ecosystem due to their in situ nature.

## Supporting information

S1 Table Ingredients and chemical composition of pooled forage-only diet and pooled high-concentrate diet. (DOCX)
