# Correction: Temporal dynamics of in-situ fiber-adherent bacterial community under ruminal acidotic conditions determined by 16S rRNA gene profiling

**DOI:** 10.1371/journal.pone.0204600

**Published:** 2018-09-20

**Authors:** Renee M. Petri, Poulad Pourazad, Ratchaneewan Khiaosa-ard, Fenja Klevenhusen, Barbara U. Metzler-Zebeli, Qendrim Zebeli

The images for Figs 1 and 4 are incorrectly switched. The image that appears as Fig 1 should be Fig 4, and the image that appears as Fig 4 should be Fig 1. The images for Figs 2 and 3 are incorrectly switched. The image that appears as Fig 2 should be Fig 3, and the image that appears as Fig 3 should be Fig 2. The figure captions appear in the correct order. Please view the corrected figures and figure captions here.

**Fig 1:**
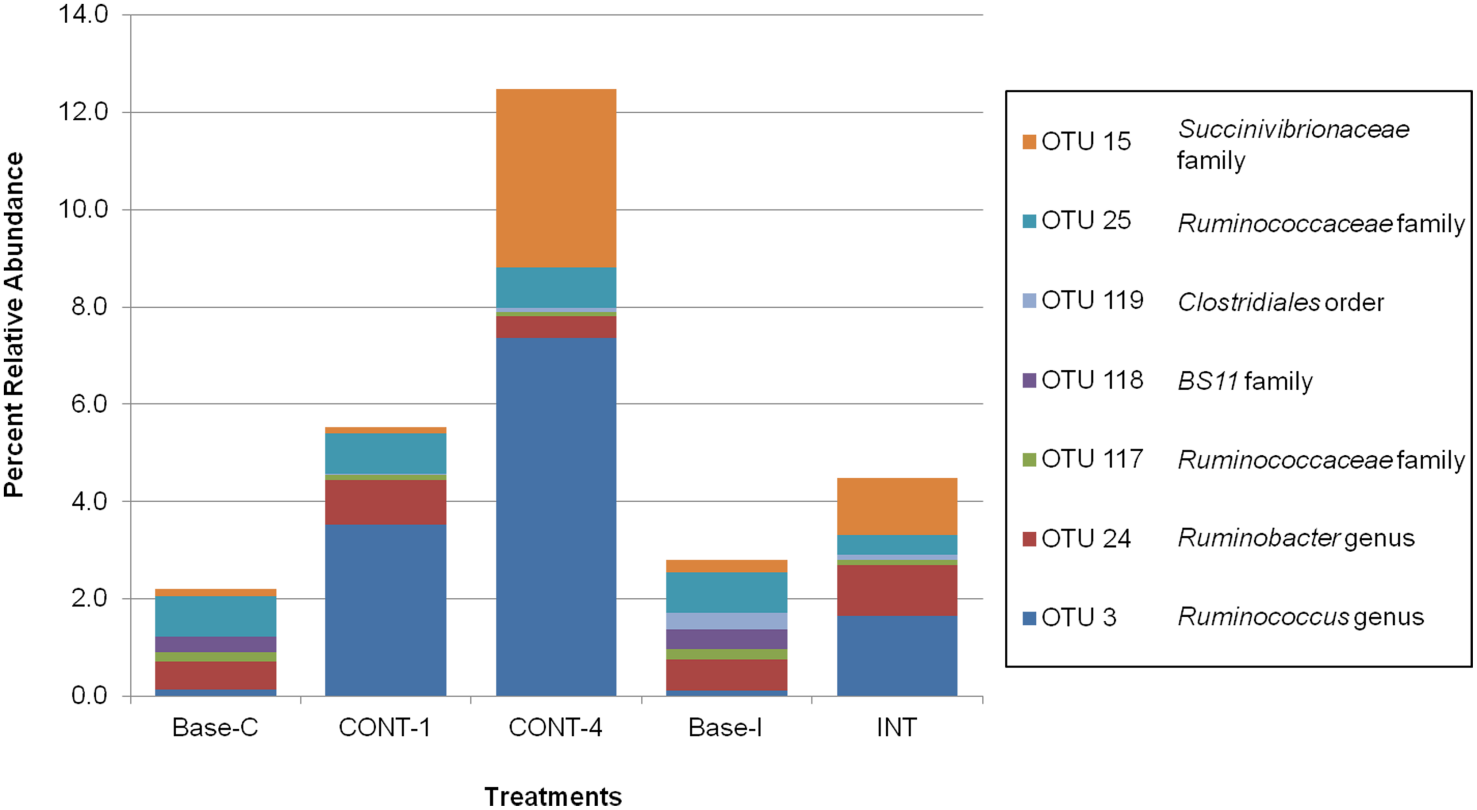
Mean values of OTUs combined with significant variation between continuous and interrupted challenge models.

**Fig 2:**
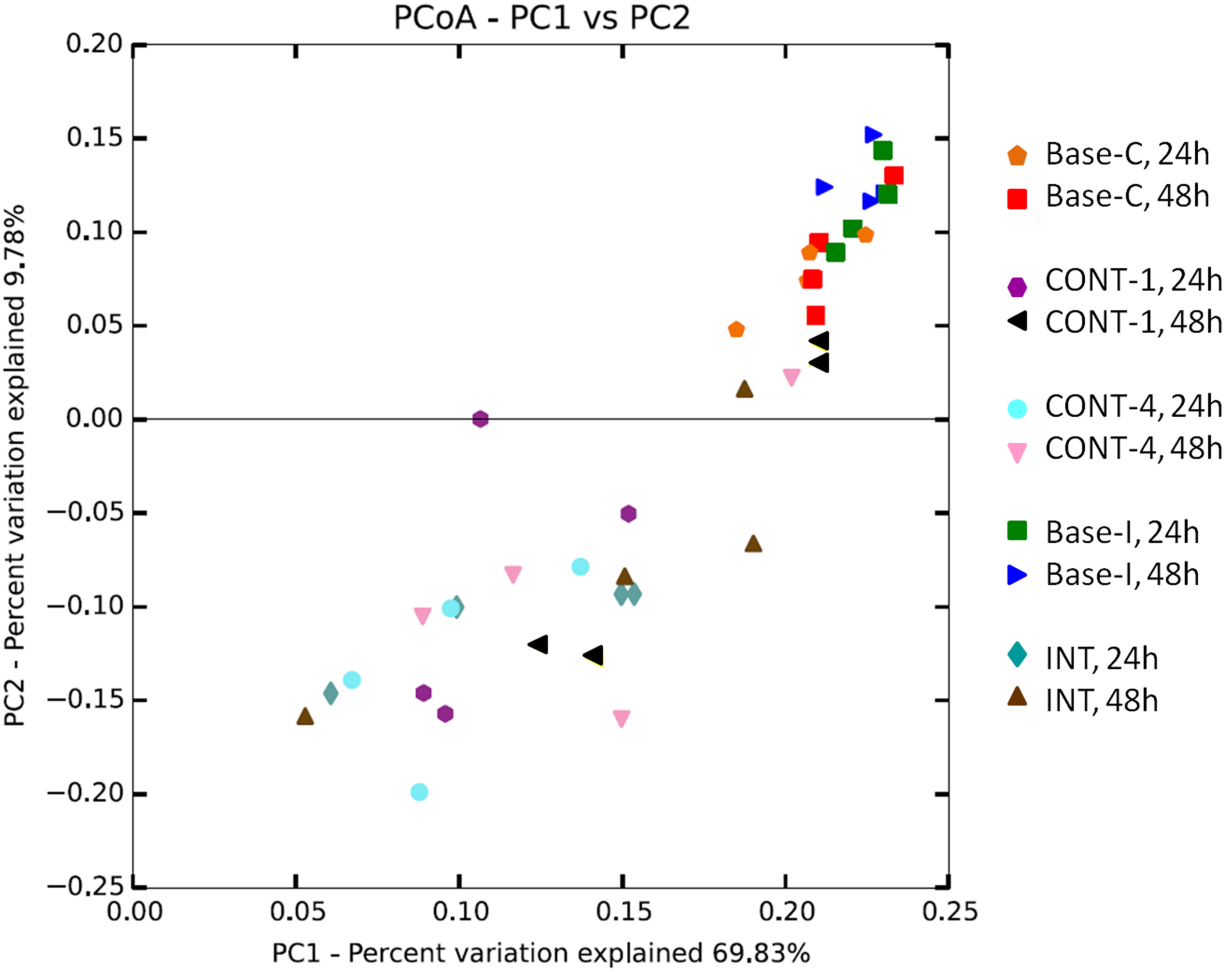
Principal coordinate analysis of the beta-diversity of rumen in-situ samples using weighted UniFrac. Analysis by PERMANOVA revealed a diet effect (P = 0.001) and an effect of feeding phase (P = 0.03).

**Fig 3:**
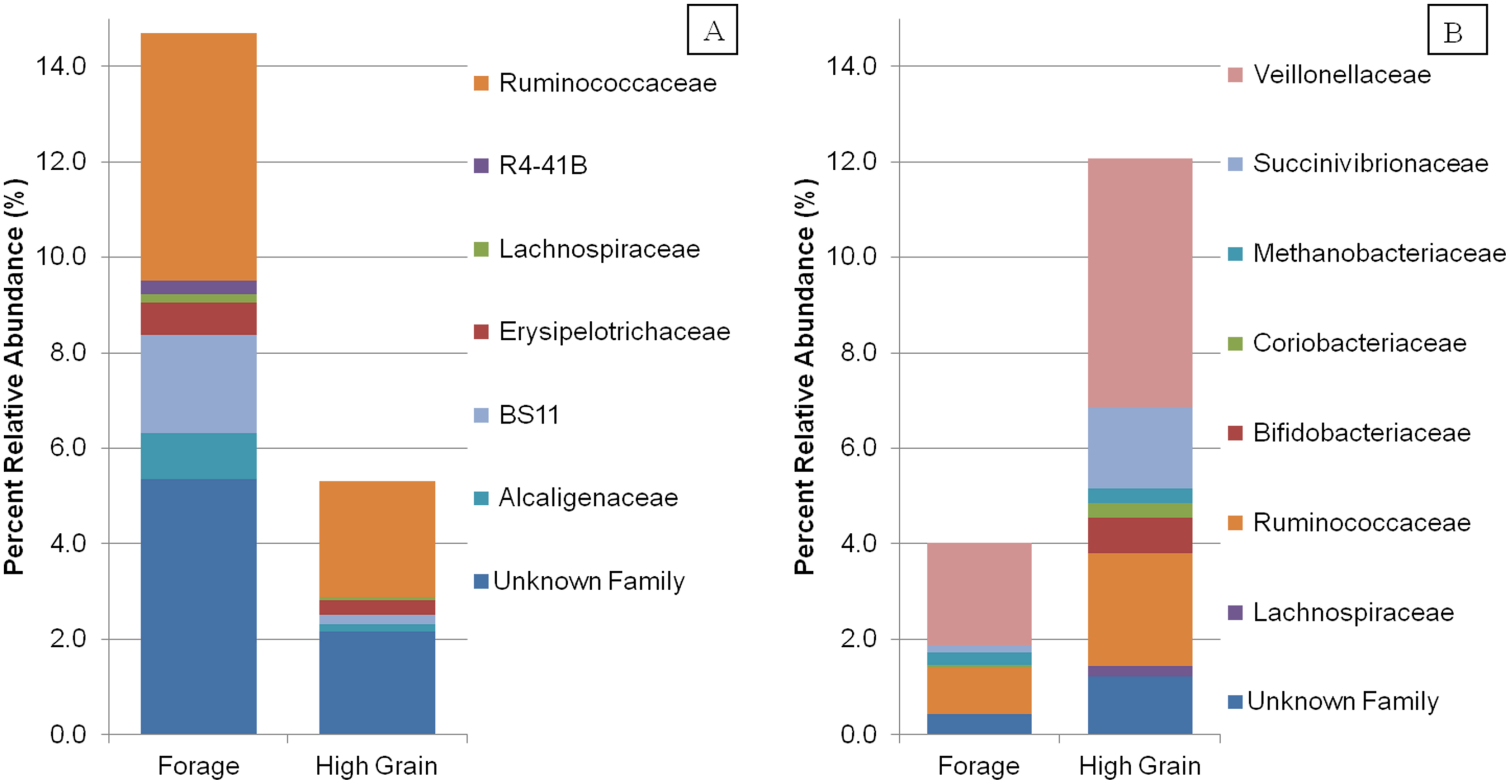
Mean percent relative abundance of family groups between diets. (A) Decreasing percent abundance (B) Increasing percent abundance in the SARA diet in comparison to the forage diet.

**Fig 4:**
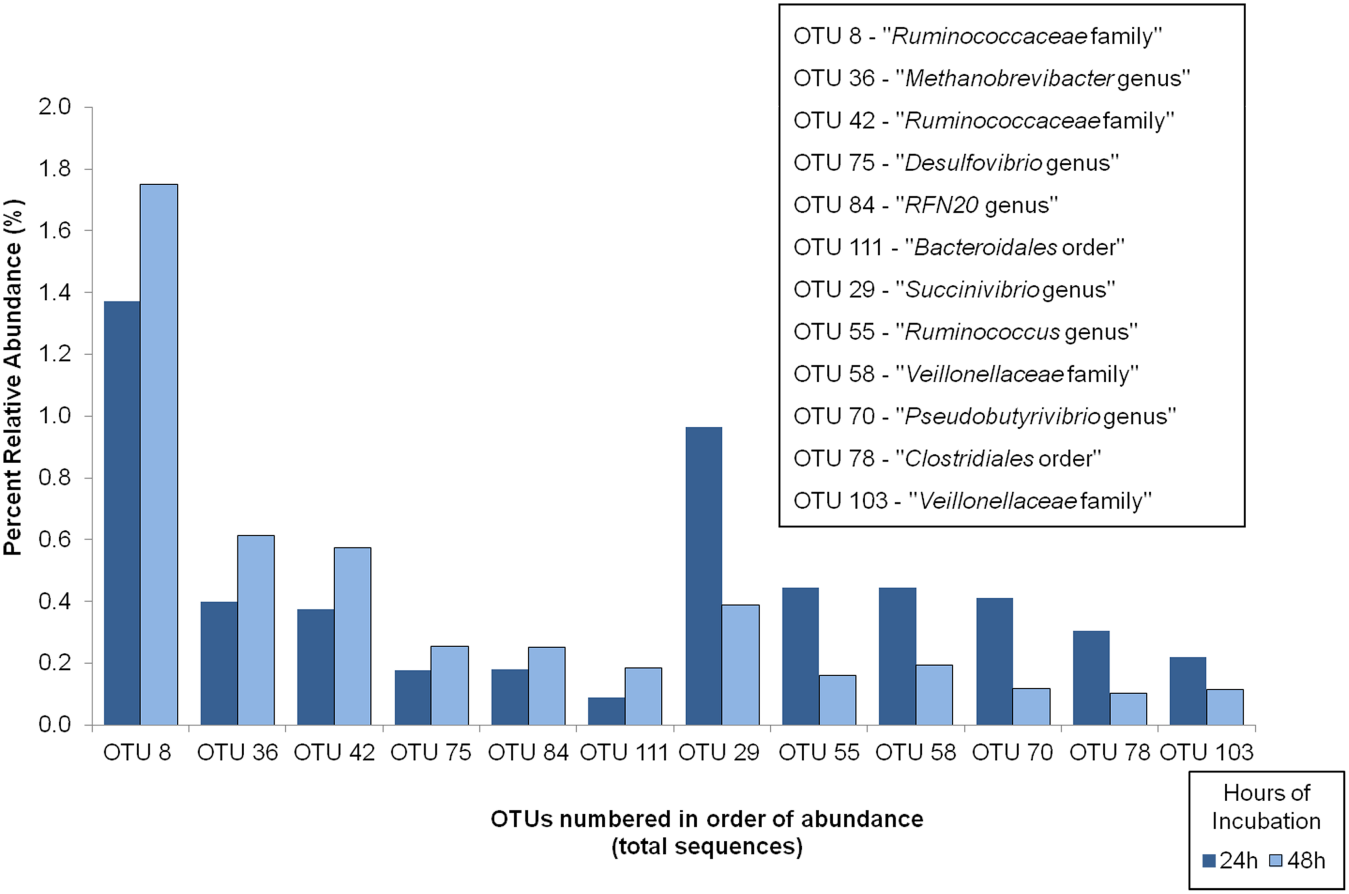
Effect of in situ incubation time on OTUs classified by the GreenGenes database.

In the second paragraph under the subheading “Microbial diversity and phylogenetic analysis” in the Results section, the fourth sentence should have cited reference 22 instead of 21.

The sentence should read: These OTUs were then secondary classified to the National Center for Biotechnology Information (NCBI) nucleotide database using Blastn for taxonomic classification and percent similarity with limitation to the 16S rRNA target (Table 3) [22].

Reference 21 was incorrectly added to the Reference list and should not be cited in the text. As a result, the in-text citations for references 22–26 are incorrect and should be cited as references 23–27.

In the second paragraph under the subheading “Microbial diversity and phylogenetic analysis” in the Results section, the seventh through tenth sentences should read: *Sporobacter* is a gram positive, obligate anaerobe identified from the gut microbiome of termites [23]. *Flintibacter butyricus* is a novel gut microbe recently identified as a major butyrate producer which ferments amino acids glutamine and glutamate [24]. The lowest percent similarity for classification was 84% for both OTU 46 (*Endomicrobia*) and OTU 23 (*RFN20*). '*Endomicrobia*', is a distinct and diverse group of uncultivated bacteria represented so far only by intracellular symbionts of termite gut flagellates. Zheng et al. [25] reported the isolation and characterization of the first free-living member of this clade from sterile-filtered gut homogenate of defaunated (starch-fed) termites. The genus *RFN20* is a rumen specific genus from the *Tenericutes* phylum [26].

In the seventh paragraph under the subheading “Microbial diversity and phylogenetic analysis” in the Results section, the first sentence should read: Pearson correlation of OTUs representing a percent relative abundance of 0.5% or greater were analyzed with relation to the environmental parameters DMI, mean pH and the duration of time spend under pH 5.8, as a reference point for environmental effects on rumen cellulolytic populations [27].
