# Supplementary material for: Temporal dynamics of in-situ fiber-adherent bacterial community under ruminal acidotic conditions determined by 16S rRNA gene profiling
Source: PLoS One. 2017 Aug 1;12(8):e0182271. doi: 10.1371/journal.pone.0182271 (PMC5538656; doi:10.1371/journal.pone.0182271)
Supplement: S1 Table — (DOCX) [file pone.0182271.s001.docx]

**Supplemental Information**

S1 Table. Ingredients and chemical composition of pooled forage-only diet and pooled high-concentrate diet

| Item | Forage only^1^ | SARA |
| --- | --- | --- |
| Forage, % of DM |  |  |
| Grass silage | 50 | 20 |
| Second-cut meadow hay | 50 | 20 |
| Concentrate, % of DM^2^ |  |  |
| Barley grain | 0 | 19.8 |
| Wheat | 0 | 18 |
| Corn | 0 | 9 |
| Rapeseed meal | 0 | 10.2 |
| Dried beet pulp | 0 | 1.9 |
| Calcium carbonate | 0 | 0.3 |
| NaCl | 0 | 0.2 |
| Mineral–vitamin premix^3^ | 0 | 0.6 |
| Chemical composition, % of DM (unless otherwise stated) | | |
| DM, % | 54.4 | 74.5 |
| OM | 91.6 | 94.1 |
| CP | 12.8 | 15.4 |
| NDF | 51.7 | 31.8 |
| ADF | 36.2 | 19.9 |
| Ether extract | 1.5 | 1.71 |
| Ash | 8.36 | 5.86 |
| NFC^4^ | 25.6 | 45.2 |
| ^1^During the baseline and concentrate break period. | | |
| ^2^Concentrate contained 88.0% DM, 95.8% OM, 17.2% CP, 1.9% ether extract, and 19.5% NDF (DM basis). | | |
| ^3^Mineral–vitamin premix contained (per kg feed) 220 g Ca, 60 g P, 30g Mg, 60 g Na, 3 g Zn, 5 g Mn, 0.01 g I, 0.04 g Se, 0.03 g Co, 0.75 g Cu,600,000 IU vitamin A, 80,000 IU vitamin D, and 2 g vitamin E. Cows were offered free access to mineral licking stones (RINDAMIN LECKSTEIN;Schaumann GmbH & Co KG, Brunn, Austria) throughout the experiment. | | |
| ^4^NFC = nonfiber carbohydrate: 100 – (ash – CP – NDF – ether extract). | | |
